# Meeting the Shared Goals of a Student-Selected Component: Pilot Evaluation of a Collaborative Systematic Review

**DOI:** 10.2196/39210

**Published:** 2023-03-15

**Authors:** Faheem Bhatti, Oliver Mowforth, Max Butler, Zainab Bhatti, Amir Rafati Fard, Isla Kuhn, Benjamin M Davies

**Affiliations:** 1 School of Clinical Medicine University of Cambridge Cambridge United Kingdom; 2 Division of Neurosurgery Department of Clinical Neurosciences University of Cambridge Cambridge United Kingdom; 3 School of Clinical Medicine University of Nottingham Medical School Nottingham United Kingdom; 4 Cambridge University Medical Library Cambridge United Kingdom

**Keywords:** medical education, medical student, research training, research methodology, systematic review, methodology, review, collaboration, collaborative, medical school, medical librarian, library science, information science, search strategy, student-selected component, curriculum, curricula

## Abstract

**Background:**

Research methodology is insufficiently featured in undergraduate medical curricula. Student-selected components are designed to offer some research opportunities but frequently fail to meet student or supervisor expectations, such as completion or publication. We hypothesized that a collaborative, educational approach to a systematic review (SR), whereby medical students worked together, may improve student experience and increase success.

**Objective:**

This study aimed to establish whether offering a small team of students the opportunity to take part in the screening phase of SRs led by an experienced postgraduate team could enhance the learning experience of students, overcome the barriers to successful research engagement, and deliver published output.

**Methods:**

Postgraduate researchers from the University of Cambridge led a team of 14 medical students to work on 2 neurosurgical SRs. One student was appointed as the lead for each SR. All students were provided with training on SR methodology and participated in title and abstract screening using Rayyan software. Students completed prepilot, midscreening, and postscreening questionnaires on their research background, perceptions, knowledge, confidence, and experience. Questions were scored on a Likert scale of 1 (strongly disagree) to 10 (strongly agree).

**Results:**

Of the 14 students involved, 29% (n=4) reported that they had received sufficient training in research methodology at medical school. Positive trends in student knowledge, confidence, and experience of SR methodology were noted across the 3 questionnaire time points. Mean responses to “I am satisfied with the level of guidance I am receiving,” “I am enjoying being involved in the SR process,” and “I could not gain this understanding of research from passive learning e.g., textbook or lecture” were greater than 8.0 at all time points. Students reported “being involved in this research has made me more likely to do research in the future” (mean 8.57, SD 1.50) and that “this collaborative SR improved my research experience” (mean 8.50, SD 1.56).

**Conclusions:**

This collaborative approach appears to be a potentially useful method of providing students with research experience; however, it requires further evaluation.

## Introduction

In *Outcomes for Graduates* [1], the General Medical Council states that medical graduates should be able to apply the scientific method and understanding of medical research when making decisions regarding patient care. Opportunities for medical students to be involved in research are now required by all medical schools in the United Kingdom. This commonly takes the form of student-selected components (SSCs), a dedicated period in the medical course where medical students can engage in a diverse range of research opportunities [1,2].

Medical students are not always able to seize the full potential of SSCs due to several factors. First, teaching in research methodology is inconsistent among undergraduate medical curricula [3]. Second, the duration of an SSC is relatively short for a project to be completed [4,5]. Limited prior research training and difficulty identifying a manageable project with good mentors provide further challenges for those with little prior research experience [3,6]. Together, these factors can leave medical students feeling poorly prepared, overwhelmed, and insufficiently supported, which can ultimately lead to a poor experience of research and eventually disengagement [7,8].

Review articles are the most common article type published by medical students [9,10]. Systematic reviews (SRs) combine a high likelihood of publication with the ability to actively contribute to research, allowing students to acquire fundamental research and evidence-based medicine skills [11,12]. As part of a quality improvement initiative, we hypothesized that a collaborative approach to SR may offer a solution to these problems. We aimed to explore whether offering a small team of students the opportunity to take part in the title and abstract screening phase of SRs while being led by an experienced postgraduate team could enhance the learning experience, overcome the barriers to successful research engagement, and deliver published output.

## Methods

### SR Conception

In all, 2 SR articles were devised by postgraduate researchers based on the current research interests of the Degenerative Cervical Myelopathy (DCM) Research Group in Cambridge, United Kingdom. Both SRs were in due reference to the priorities of patients with DCM, expressed through forums including *Myelopathy.org*, an international myelopathy charity, and the Research Objectives and Common Data Elements for DCM process, an international consensus process to define the research priorities for DCM [13-15]. The topics of the reviews were (1) the impact of phosphodiesterase 3 and 4 inhibition on neurobehavioral outcomes in preclinical models of traumatic and nontraumatic spinal cord injury and (2) the role of cannabinoids on modulating neurobehavioral outcomes in preclinical models of traumatic and nontraumatic spinal cord injury [16]. Both reviews were registered on PROSPERO (University of York, United Kingdom; CRD42019150639 and CRD42019149671, respectively). Search strategy and protocol development was led by the 2 lead students, with reference to previous SRs conducted by our group, followed by review, discussion, and feedback from postgraduate researchers [15-21].

### Recruitment

A national advertisement was disseminated by the national network of the Myelopathy.org Student Society to recruit medical student and junior doctors interested in participating in the title and abstract screening phase of the SRs. A total of 14 students applied to be involved. All 14 students were invited to participate to promote inclusivity given the flexibility in the number of students that could be involved.

An undergraduate medical student was selected to lead each review under the supervision of postgraduate researchers and a medical librarian at the University of Cambridge.

### Collaborative Process

Postgraduate researchers provided the 14 students with training, including written guidance, on the process of title and abstract screening, in addition to search strategy and inclusion and exclusion criteria formulation. All students were given the opportunity to email questions, and explanations were provided. Rayyan software (Rayyan Systems) was used to enable a collaborative multiresearcher approach to the screening of titles and abstracts, ensuring that each article was independently reviewed by 2 students [22]. Initially, a Rayyan sandbox containing a pilot sample of 100 titles and abstracts was created. All 14 students screened the 100 titles and abstracts. The student pilot-screening results were then compared to those of the postgraduate researchers. Subsequently, definitions were clarified and explanatory statements for the inclusion and exclusion criteria were revised to ensure strong interstudent reliability.

The 14 students were then equally involved in completing title and abstract screening for the 2 SRs. A total of 10,251 titles and abstracts were allocated (8714 and 1537 articles from the 2 SRs) such that each title and abstract was screened by 2 students. This resulted in each student screening 1464 articles. Following the completion of screening, the 2 leading undergraduate students then completed the remainder of the SRs. As a pilot evaluation of this approach, this was a pragmatic decision, given the uncertainty of the effectiveness of the collaborative approach. The remaining 12 students were updated on project progress and provided with written materials on the key stages of SR, in addition to specific examples from the present SRs.

### Survey Design

**Figure 1:**
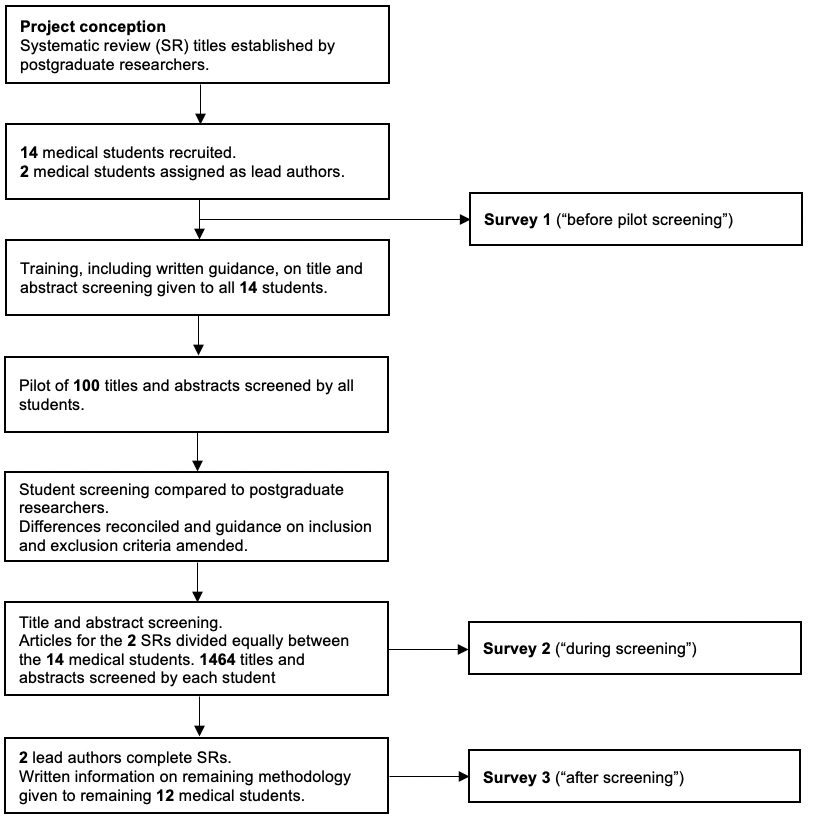
Summary of project methodology.

To enable the assessment of the effectiveness of this methodology, participating students completed 3 surveys throughout the process. The first survey was conducted prior to the pilot screening of 100 articles, the second after the completion of pilot screening and during screening of the titles and abstracts for the 2 SRs, and the third after the completion of all title and abstract screening and the provision of the written summary of the remaining SR methodology. Figure 1 illustrates the timings of the surveys. All 3 surveys assessed students’ perceptions of research; experience of this collaborative initiative; and their “knowledge,” “confidence,” and “experience” of SR methodology. SR methodology was divided into 12 components: question formulation, development of a search strategy, development of inclusion and exclusion criteria, title and abstract screening, full-text screening, risk of bias assessment, development of an extraction template, data extraction, data synthesis, data interpretation, manuscript writing, and presentation skills. In addition, the first survey captured information such as the stage of training, prior research experience, and the amount of research methodology teaching received.

In total, there were 85 questions across the 3 surveys. Of these, 67 questions were close ended in Likert-scale format with a scale from 0 to 10, with 0 being “strongly disagree,” 5 being “neither agree or disagree,” and 10 being “strongly agree.” The full list of questions in each survey is available in Multimedia Appendix 1. The questionnaires were hosted using the SurveyMonkey platform (Momentive Inc). Each student created a unique identifier that was entered each time they completed a survey to allow changes in perceptions to be anonymously measured over time. Reminders for survey completion were sent to students throughout the process; however, survey completion remained voluntary.

### Data Analysis

Survey results were exported into Microsoft Excel, where responses were collated. Descriptive statistics, including means and SDs, were calculated where appropriate. Inferential statistical analysis was not appropriate given the small sample size of students (N=14).

### Ethical Considerations

Ethical approval was not obtained as this project was considered an initial part of a quality improvement process looking to improve student experience of SSCs. The findings are intended to inform the optimization of a teaching program that would still need subsequent evaluation. This was checked with the Human Research Authority, using their decision aid [23] to arrive at this conclusion.

## Results

### Response Rates

All 14 students responded to each of the 3 surveys, answering all the questions apart from 2 questions where 1 student did not respond (questions assessing the experience of full-text screening and experience of manuscript writing).

### Student Demographics and Prior Research Experience

Demographics and previous research experience are summarized in Table 1 (see Multimedia Appendix 1 for additional information). When asked what specialties they were interested in, 10 (71%) out of 14 students expressed interest in neurology or neurosurgery, and 10 (71%) considered research to be necessary to secure a training post in their desired specialty.

**Table 1:** Student demographics and previous research experience.

Demographic or experience and response			Student (N=14), n (%)
**Sex**			
	Male	8 (57)	
	Female	6 (43)	
**Age (years)**			
	≤21	5 (36)	
	22-25	4 (29)	
	≥26	5 (36)	
**Year of study**			
	3	2 (14)	
	4	7 (50)	
	5	2 (14)	
	6	2 (14)	
	Foundation year 1 doctor	1 (7)	
**Previous completed degrees**			
	Bachelor’s level	5 (36)	
	Master’s level	3 (21)	
**Previously been an author of a PubMed-indexed systematic review**			
	Yes	2 (14)	
	No	12 (86)	
**Previously published a first-author publication in a PubMed-indexed journal**			
	Yes	3 (21)	
	No	11 (79)	
**Previously published a non–first-author publication in a PubMed-indexed journal**			
	Yes	3 (21)	
	No	11 (79)	
**Previously presented research at national or international conferences**			
	Yes	8 (57)	
	No	6 (43)	

### Research Methodology Teaching Received

A summary of the amount and form of research methodology teaching students received and their perceptions are provided in Table 2. The most common form of teaching was lectures (6/14, 43%). Of the 14 students, 4 (29%) agreed with the statement, “I have had sufficient training in research methodology at medical school”; whereas 2 (14%) students strongly agreed and 5 (36%) students agreed with the statement, “I have had sufficient opportunity to participate in research at medical school.”

**Table 2:** Research methodology teaching received.

Question and response			Student (N=14), n (%)
**Hours of mandatory teaching on research methodology received at university?**			
	None	0 (0)	
	<2 hours	4 (29)	
	2-5 hours	3 (21)	
	5-10 hours	3 (21)	
	>10 hours	4 (29)	
**Hours of voluntary/extra-curricular teaching on research methodology attended at university?**			
	None	4 (29)	
	<2 hours	3 (21)	
	2-5 hours	4 (29)	
	5-10 hours	1 (7)	
	>10 hours	2 (14)	
**Form of research teaching**			
	Lecture	6 (43)	
	Seminar	3 (21)	
	Tutorial	2 (14)	
	Other	3 (21)	
**To what extent do you agree with the following statement: I have had sufficient training in research methodology at medical school.**			
	Strongly agree	0 (0)	
	Agree	4 (29)	
	Neutral	5 (36)	
	Disagree	3 (21)	
	Strongly disagree	2 (14)	
**To what extent do you agree with** **the following statement: I have had sufficient opportunity to participate in research at medical school.**			
	Strongly agree	2 (14)	
	Agree	5 (36)	
	Neutral	3 (21)	
	Disagree	3 (21)	
	Strongly disagree	1 (7)	

### How Did Perceptions of Research Change Throughout the Process?

Table 3 summarizes how perceptions of research changed during the collaborative SR training process. There were increases in the responses to “I am good at research,” “I am confident at research,” “I am experienced at research,” “I have experience conducting systematic reviews,” “I am confident with the theory of a systematic review,” and “I am confident with the practicalities of conducting a systematic review.” There was otherwise little change in the perceptions of the other statements. The average response to “I enjoy research” and “Research is interesting” in the prepilot survey was 8.07 (SD 1.59) and 8.21 (SD 1.88), respectively. Similarly, the average response to “I would consider being involved in research in the future” was greater than or equal to 9 in all 3 surveys.

**Table 3:** Responses to questions assessing research perceptions at 3 time points.

	Prepilot, mean (SD)	During screening, mean (SD)	After screening, mean (SD)
I enjoy research	8.07 (1.59)	7.79 (2.12)	8.36 (1.69)
I am good at research	6.29 (1.77)	6.43 (1.83)	7.07 (1.69)
I am confident conducting research	5.43 (2.56)	6.50 (1.79)	7.07 (1.77)
I am experienced at research	4.86 (2.44)	6.07 (1.54)	6.64 (1.44)
Research is interesting	8.21 (1.89)	8.07 (2.02)	8.79 (1.85)
Research is important	10.00 (0.00)	9.5 (0.76)	9.79 (1.58)
Research is difficult	7.21 (1.37)	6.36 (1.50)	6.21 (1.31)
Research is best left to scientists and/or senior doctors	2.86 (2.11)	2.64 (2.98)	2.71 (2.23)
I would consider being involved in research in the future	9.29 (0.99)	9.00 (1.24)	9.57 (0.94)
I have experience conducting systematic reviews	3.86 (3.74)	5.43 (2.28)	6.71 (1.98)
I am confident with the theory of a systematic review	6.21 (2.78)	7.00 (1.47)	7.64 (1.08)
I am confident with the practicalities of conducting a systematic review	5.21 (3.26)	6.57 (2.21)	7.36 (1.86)

### How Did Knowledge, Confidence, Experience of SR Methodology Change Throughout the Process?

**Figure 2:**
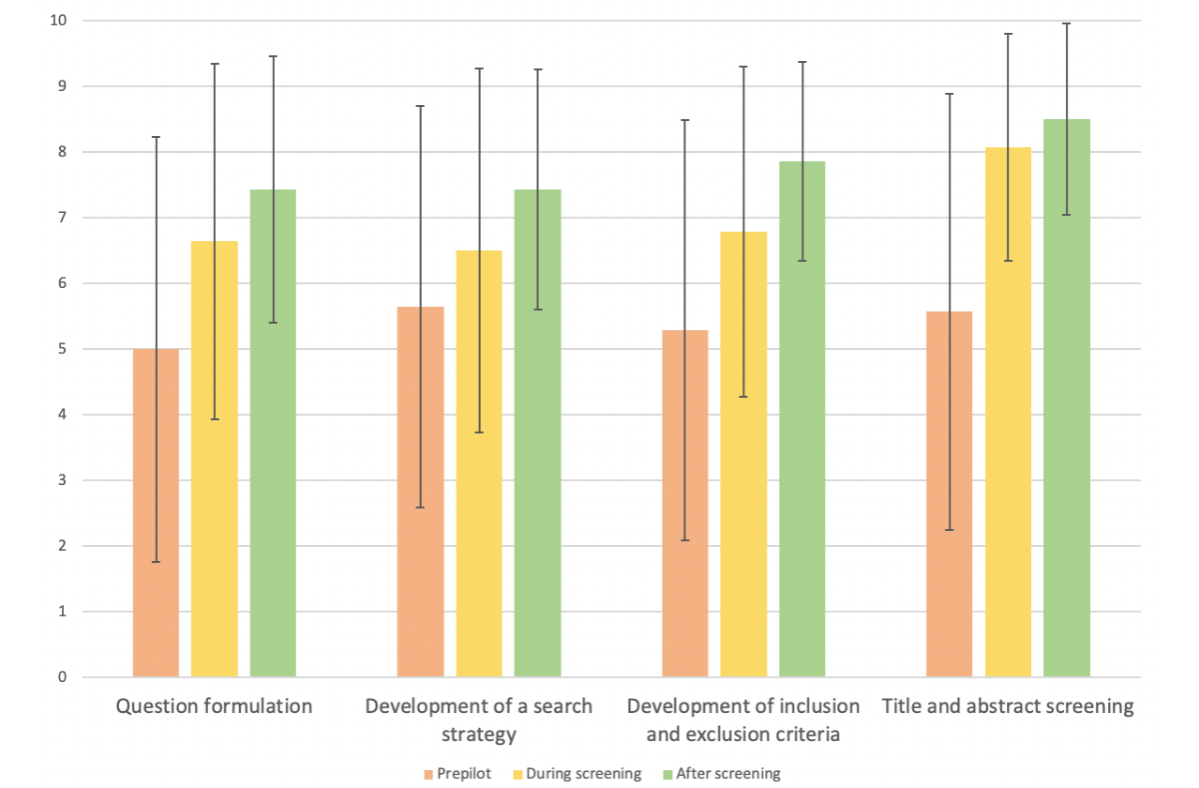
How the knowledge of systematic review methodology changed throughout the process (mean and SD).

**Figure 3:**
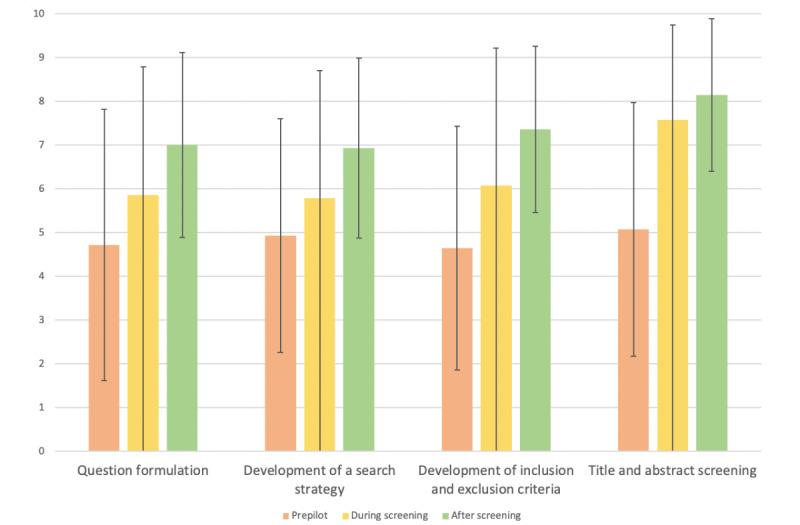
How the confidence of systematic review methodology changed throughout the process (mean and SD).

**Figure 4:**
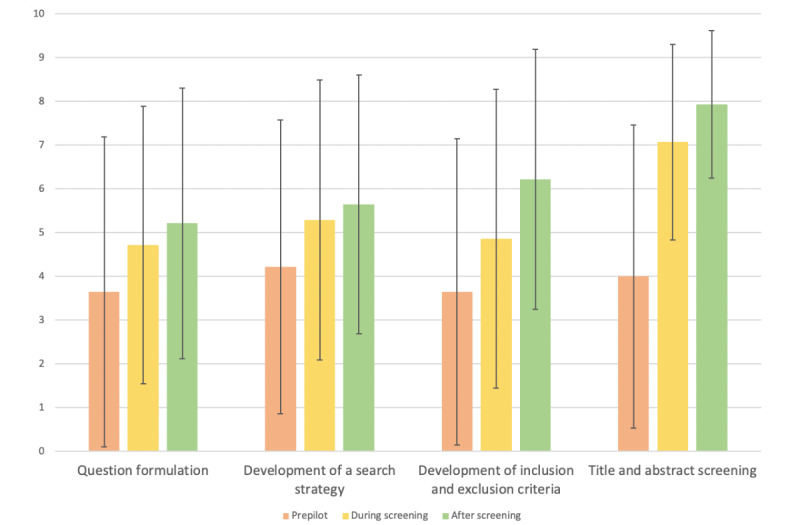
How the experience of systematic review methodology changed throughout the process (mean and SD).

Tables 4-6 and Figures 2-4 illustrate how knowledge, confidence, and experience of the 12 components of SR methodology changed before, during, and after title and abstract screening. An increase in mean scores of knowledge, confidence, and experience of all 12 components was noted in the postscreening survey compared to the prepilot survey. The largest increases in knowledge (before: mean 5.57, SD 3.32 vs after: mean 8.50, SD 1.45), confidence (before: mean 5.07, SD 2.89 vs after: mean 8.14, SD 1.75), and experience (before: mean 4.00, SD 3.46 vs after: mean 7.93, SD 1.69) across the process were noted for title and abstract screening.

**Table 4:** Knowledge of systematic review methodology assessed at 3 time points.

	Prepilot, mean (SD)	During screening, mean (SD)	After screening, mean (SD)
Question formulation	5 (3.23)	6.64 (2.71)	7.42 (2.03)
Development of a search strategy	5.64 (3.05)	6.50 (2.77)	7.43 (1.83)
Development of inclusion and exclusion criteria	5.29 (3.20)	6.79 (2.52)	7.86 (1.51)
Title and abstract screening	5.57 (3.32)	8.07 (1.73)	8.5 (1.45)
Full-text screening	5.29 (3.31)	5.57 (2.90)	6.86 (2.60)
Risk of bias assessment	3.86 (3.08)	4.14 (2.38)	5.36 (2.56)
Development of an extraction template	3.36 (3.18)	3.00 (2.72)	3.86 (2.93)
Data extraction	4.00 (3.33)	3.71 (2.97)	5.00 (3.01)
Data synthesis	3.79 (3.02)	3.42 (3.00)	5.07 (2.79)
Data interpretation	5.21 (3.14)	4.86 (3.25)	6.07 (2.89)
Manuscript writing	5.36 (3.39)	5.57 (3.41)	6.29 (3.10)
Presentation skills	6.00 (3.42)	6.21 (2.91)	6.71 (2.95)

**Table 5:** Confidence in systematic review methodology assessed at 3 time points.

	Prepilot, mean (SD)	During screening, mean (SD)	After screening, mean (SD)
Question formulation	4.71 (3.10)	5.86 (2.93)	7 (2.11)
Development of a search strategy	4.93 (2.67)	5.79 (2.91)	6.93 (2.06)
Development of inclusion and exclusion criteria	4.64 (2.79)	6.07 (3.15)	7.36 (1.91)
Title and abstract screening	5.07 (2.89)	7.57 (2.17)	8.14 (1.75)
Full-text screening	4.64 (2.98)	5.14 (2.85)	6.64 (2.71)
Risk of bias assessment	3.21 (2.52)	3.93 (2.23)	4.71 (2.52)
Development of an extraction template	3.29 (2.81)	3.14 (2.54)	4.29 (2.89)
Data extraction	4.14 (3.03)	4.00 (2.94)	5.07 (2.79)
Data synthesis	3.86 (3.03)	4.21 (2.83)	5.14 (2.93)
Data interpretation	5.14 (3.08)	5.07 (2.64)	6.21 (2.67)
Manuscript writing	5.29 (3.20)	5.29 (3.02)	6.36 (2.84)
Presentation skills	5.71 (3.10)	6.00 (2.96)	6.86 (2.93)

**Table 6:** Experience of systematic review methodology assessed at 3 time points.

	Prepilot, mean (SD)	During screening, mean (SD)	After screening, mean (SD)
Question formulation	3.64 (3.54)	4.71 (3.17)	5.21 (3.09)
Development of a search strategy	4.21 (3.26)	5.29 (3.20)	5.64 (2.95)
Development of inclusion and exclusion criteria	3.64 (3.50)	4.86 (3.42)	6.21 (2.97)
Title and abstract screening	4.00 (3.46)	7.07 (2.23)	7.93 (1.69)
Full-text screening	4.15 (3.56)^a^	4.36 (3.50)	5.29 (2.89)
Risk of bias assessment	3.00 (2.88)	2.79 (2.29)	3.29 (2.70)
Development of an extraction template	2.79 (2.83)	2.79 (2.89)	3.36 (2.98)
Data extraction	3.50 (3.23)	3.79 (3.24)	4.21 (2.94)
Data synthesis	3.43 (2.95)	4.00 (3.28)	4.64 (2.73)
Data interpretation	4.57 (2.95)	4.79 (3.47)	5.14 (3.03)
Manuscript writing	4.86 (3.21)	5.00 (3.58)^a^	5.93 (3.10)
Presentation skills	5.29 (3.43)	5.64 (3.52)	6.50 (3.03)

^a^Only 13 responses to these questions were received.

### Evaluation of the Process

**Figure 5:**
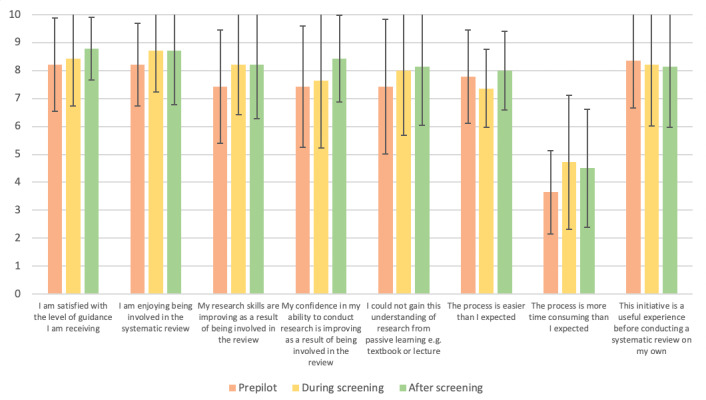
Evaluation of the collaborative process (mean and SD).

Figure 5 highlights student students’ evaluation of the collaborative process across the 3 time points. Additional questions were asked in the final survey, and the mean responses to these statements are as follows: “This collaborative SR improved my research experience” (mean 8.50, SD 1.56), “My understanding of research methodology improved as a result of being part of this review” (mean 7.64, SD 1.86), “Being involved in this research has made me more likely to do research in the future” (mean 8.57, SD 1.50), and “Being involved in this research has made me more likely to do myelopathy research in the future” (mean 7.2, SD 2.39).

When asked whether the “Overall experience was worthwhile,” all 14 (100%) students responded “yes.” When asked, “Would you have preferred to be involved in all stages of the review?” 11 (79%) of the 14 students responded “yes.”

### Research Output

As of the time of writing, 1 of the SRs has been published and the other is being prepared for submission [16].

## Discussion

### Principal Findings

Our study provides insight into the perspectives of medical students involved in a trial of a collaborative approach to SR, in which students were given the opportunity to be involved in research while being closely supported by experienced postgraduate clinical researchers. Within the practical limitations of students primarily being involved in title and abstract screening, the responses to our questionnaires suggest the approach was well received by those involved.

With regard to prior understanding of research methodology, the questionnaire identified that the teaching of research methodology received by students varied in format and quantity. All students involved received at least some form of teaching on research methodology at university; however, only 29% of students agreed that the teaching they received was of sufficient quantity. This finding is in alignment with a larger questionnaire of medical students, which found that 43% of respondents felt their medical school provided adequate research training [3]. SSCs present students with a learning opportunity to gain insight into research that may not have been provided through medical school lectures, seminars, or tutorials.

The collaborative approach appeared to be useful in introducing students to research. A negative trend in the perceived difficulty of research was observed across the 3 questionnaires, which could suggest that a collaborative approach, such as this one, may be helpful in making research more accessible for medical students. Positive trends in self-reported knowledge, confidence, and experience of SR methodology were also noted. The biggest changes in knowledge, confidence, and experience were for the process of title and abstract screening. This was the process that the medical students were actively involved in and gained hands-on experience of. Active learning in which students have opportunities to participate and engage with their learning is supported by adult learning theory and is being increasingly used in medical education [24,25]. Furthermore, students reported that the understanding of research they obtained from being involved in this program could not have been obtained from “passive learning e.g., textbook or lecture.” Given that this was an initial trial of this collaborative approach to SRs, students were primarily involved in title and abstract screening. Future projects involving greater student participation, for example, in data extraction, may prove useful in further elucidating the efficacy of collaborative approaches to SRs.

It has previously been shown that poor initial experiences with research can lead to disengagement [7,8]. On the other hand, positive experiences of research with good mentorship are associated with increased interest in research and future research participation [26,27]. The benefits of successful research engagement are not limited to research and academia [5]. Research placements provide an opportunity for medical students to gain deeper insight into a specialty of their choosing, thus placing them in a position to make informed career choices [26]. Students have been shown to be 2.7 times more likely to pursue the same clinical specialty that they undertook a research project in while at medical school [5,28]. These factors emphasize the significance of the initial exposure to research that medical students experience, both in terms of their future clinical practice and scientific output. Throughout the collaborative process, levels of enjoyment and satisfaction with the level of guidance were consistently high. Additionally, students responded positively to the statement, “being involved in this research has made me more likely to do research in the future,” with a mean response of 8.57 (SD 1.50) out of 10. Although the students in this study were primarily only involved in title and abstract screening, a collaborative approach may be an enjoyable method of involving students in research.

### Limitations

First, as this was a pilot evaluation of the collaborative approach to SR, this study was conducted by 1 research group and involved a small number of medical students (N=14) working on title and abstract screening for 2 SRs. This was a pragmatic decision given the uncertainty regarding the efficacy of the approach. Due to this small sample size, inferential statistics were not considered appropriate. Following this pilot, future studies should involve multiple research groups, with larger numbers of students, and student participation in a greater proportion of the SR process to better evaluate the collaborative approach.

Second, students were recruited from the student network of Myelopathy.org, as this was the most practical option for reaching out to medical students. This approach may have selected for students more interested in an academic career in neuroscience, which may have skewed perceptions toward research. Third, several students involved had previous degrees and research experience, potentially impacting scores of knowledge, confidence, and experience of SR methodology throughout the process. This student group is therefore unlikely to represent all medical students, and further exploration of this collaborative approach with subgroup analysis between those with and without prior research experience would be insightful.

This was ultimately an initial, small-scale exploration of whether real-world experience of a SR was advantageous to medical students. The findings of this study should therefore inform further optimization, including consideration of the aforementioned limitations, and subsequent formal evaluation.

### Conclusions

Within the limitations of the study, this collaborative and educational approach to SR was well received by medical students, allowing them to gain insight into research methodology while contributing to publishable research. This potentially represents a useful technique for SSC projects; however, it requires further formal evaluation.

## Appendix

Multimedia Appendix 1 Supplementary material containing full questionnaires and full participant demographics.

## Abbreviations

DCM: degenerative cervical myelopathy
SR: systematic review
SSC: student-selected component

## Data Availability

The data sets generated and analyzed during the current study are available from the corresponding author on reasonable request.

## References

[1] Outcomes for graduates. General Medical Council.

[2] Stark P, Ellershaw J, Newble D, Perry M, Robinson L, Smith J, Whittle Sue, Murdoch-Eaton Deborah (2005). Student-selected components in the undergraduate medical curriculum: a multi-institutional consensus on assessable key tasks. Med Teach.

[3] Funston G, Piper R, Connell C, Foden P, Young A, O'Neill Paul (2016). Medical student perceptions of research and research-orientated careers: an international questionnaire study. Med Teach.

[4] Murdoch-Eaton D, Drewery S, Elton S, Emmerson C, Marshall M, Smith J, Stark Patsy, Whittle Sue (2010). What do medical students understand by research and research skills? identifying research opportunities within undergraduate projects. Med Teach.

[5] Amgad M, Man Kin Tsui Marco, Liptrott S, Shash E (2015). Medical student research: an integrated mixed-methods systematic review and meta-analysis. PLoS One.

[6] Ranieri V, Barratt H, Fulop N, Rees G (2016). Factors that influence career progression among postdoctoral clinical academics: a scoping review of the literature. BMJ Open.

[7] InciSioN UK Collaborative (2020). Global health education in medical schools (GHEMS): a national, collaborative study of medical curricula. BMC Med Educ.

[8] Cross P (2003). Getting the most out of SSMs. BMJ.

[9] Murdoch-Eaton D, Ellershaw J, Garden A, Newble D, Perry M, Robinson L, Smith John, Stark Patsy, Whittle Sue (2004). Student-selected components in the undergraduate medical curriculum: a multi-institutional consensus on purpose. Med Teach.

[10] Wickramasinghe DP, Perera CS, Senarathna S, Samarasekera DN (2013). Patterns and trends of medical student research. BMC Med Educ.

[11] Choi AR, Cheng DL, Greenberg PB (2019). Twelve tips for medical students to conduct a systematic review. Med Teach.

[12] Droogan J, Song F (1996). The process and importance of systematic reviews. Nurse Res.

[13] Davies BM, Khan DZ, Mowforth OD, McNair AGK, Gronlund T, Kolias AG, Tetreault Lindsay, Starkey Michelle L, Sadler Iwan, Sarewitz Ellen, Houlton Delphine, Carter Julia, Kalsi-Ryan Sukhvinder, Aarabi Bizhan, Kwon Brian K, Kurpad Shekar N, Harrop James, Wilson Jefferson R, Grossman Robert, Curt Armin, Fehlings Michael G, Kotter Mark R N (2019). RE-CODE DCM (Research Objectives and Common Data Elements for Degenerative Cervical Myelopathy): a consensus process to improve research efficiency in DCM, through establishment of a standardized dataset for clinical research and the definition of the research priorities. Global Spine J.

[14] Davies B, Mowforth O, Sadler I, Aarabi B, Kwon B, Kurpad S, Harrop James S, Wilson Jefferson R, Grossman Robert, Fehlings Michael G, Kotter Mark (2019). Recovery priorities in degenerative cervical myelopathy: a cross-sectional survey of an international, online community of patients. BMJ Open.

[15] Mowforth OD, Khan DZ, Wong MY, Pickering GAE, Dean L, Magee J, Mullarkey Laura, Hirayama Yuri, Rihova Martina, Butler Max, Stewart Max, Goulson Beth, Ahmed Shahzaib, Fricke Kai, Popa-Nimigean Vladimir, Millar Zack, Venkatesh Ashwin, Willison Alice, Senthil Keerthi, Hazenbiller Olesja, Sarewitz Ellen, Sadler Iwan, Gronlund Toto, Tetreault Lindsay, Harrop James S, Aarabi Bizhan, Rahimi-Movaghar Vafa, Kurpad Shekar N, Guest James D, Wilson Jefferson R, Kwon Brian K, Fehlings Michael G, McNair Angus G K, Davies Benjamin M, Kotter Mark R N, AO Spine RECODE-DCM Steering Committee, AO Spine RECODE-DCM Consortium (2022). Gathering global perspectives to establish the research priorities and minimum data sets for degenerative cervical myelopathy: sampling strategy of the first round consensus surveys of AO spine RECODE-DCM. Global Spine J.

[16] Bhatti FI, Mowforth OD, Butler MB, Bhatti AI, Adeeko S, Akhbari M, Dilworth Rory, Grodzinski Ben, Osunronbi Temidayo, Ottewell Luke, Teh Jye Quan, Robinson Sophie, Suresh Gayathri, Waheed Unaiza, Walker Benn, Kuhn Isla, Smith Lara, Bartlett Richard D, Davies Benjamin M, Kotter Mark R N (2021). Systematic review of the impact of cannabinoids on neurobehavioral outcomes in preclinical models of traumatic and nontraumatic spinal cord injury. Spinal Cord.

[17] Grodzinski B, Durham R, Mowforth O, Stubbs D, Kotter MRN, Davies BM (2021). The effect of ageing on presentation, management and outcomes in degenerative cervical myelopathy: a systematic review. Age Ageing.

[18] Grodzinski B, Bestwick H, Bhatti F, Durham R, Khan M, Partha Sarathi Celine Iswarya, Teh Jye Quan, Mowforth Oliver, Davies Benjamin (2021). Research activity amongst DCM research priorities. Acta Neurochir (Wien).

[19] Partha Sarathi Celine I, Mowforth OD, Sinha A, Bhatti F, Bhatti A, Akhbari M, Ahmed Shahzaib, Davies Benjamin M (2021). The role of nutrition in degenerative cervical myelopathy: a systematic review. Nutr Metab Insights.

[20] Hirayama Y, Mowforth OD, Davies BM, Kotter MRN (2023). Determinants of quality of life in degenerative cervical myelopathy: a systematic review. Br J Neurosurg.

[21] Pope DH, Davies BM, Mowforth OD, Bowden AR, Kotter MRN (2020). Genetics of degenerative cervical myelopathy: a systematic review and meta-analysis of candidate gene studies. J Clin Med.

[22] Ouzzani M, Hammady H, Fedorowicz Z, Elmagarmid A (2016). Rayyan-a web and mobile app for systematic reviews. Syst Rev.

[23] Do I need NHS REC review?. Medical Research Council.

[24] Taylor DCM, Hamdy H (2013). Adult learning theories: implications for learning and teaching in medical education: AMEE Guide No. 83. Med Teach.

[25] Ramnanan CJ, Pound LD (2017). Advances in medical education and practice: student perceptions of the flipped classroom. Adv Med Educ Pract.

[26] Dorrance KA, Denton GD, Proemba J, la Rochelle J, Nasir J, Argyros G, Durning SJ (2008). An internal medicine interest group research program can improve scholarly productivity of medical students and foster mentoring relationships with internists. Teach Learn Med.

[27] Zier Karen, Friedman E, Smith L (2006). Supportive programs increase medical students' research interest and productivity. J Investig Med.

[28] Bierer SB, Chen HC (2010). How to measure success: the impact of scholarly concentrations on students--a literature review. Acad Med.

