# Supplementary material for: Meeting the Shared Goals of a Student-Selected Component: Pilot Evaluation of a Collaborative Systematic Review
Source: JMIR Med Educ. 2023 Mar 15;9:e39210. doi: 10.2196/39210 (PMC10132035; doi:10.2196/39210)
Supplement: Multimedia Appendix 1 [file mededu_v9i1e39210_app1.docx]

Supplementary Material

Table of Contents

[Survey 1 - Pre-screening questionnaire 2](#_Toc103266114)

[Survey 2 - Mid-screening questionnaire 5](#_Toc103266119)

[Survey 3 - Post-screening questionnaire 7](#_Toc103266122)

[Student Demographics 9](#_Toc103266125)

# Survey 1 - Pre-pilot questionnaire

## Part One. Your Research Background

1. Confidential ID number – to link to subsequent questionnaires.
2. Gender
3. Age
4. Year of medical school / stage of postgraduate training
5. Additional degree(s) – subject and years of study
6. Planned/most likely career speciality
7. Is research a necessary to secure a training post in you desired speciality? (Yes/No/Not Sure)
8. Modal medical school exam performance (1st/2i/2ii/3^rd^/distinction/ honours/pass)
9. On how many published PubMed-indexed systematic reviews have you been an author?
10. Current number of published first-author publications in PubMed-indexed journals
11. Current number of published non-first-author publications in PubMed-indexed journals
12. Current number of other published research publications (e.g. non-PubMed-indexed journals, student journals etc.)
13. Number of weeks laboratory research-experience
14. Number of weeks of clinical research experience
15. Number of national/international conference presentations you have given
16. Number of other research presentations (e.g. oral or poster presentations at regional/local/student conferences)
17. How many hours of mandatory teaching have you had on research methodology at university (none, less than 2 hours, 2-5 hours, 5-10 hours, >10 hours)
18. How many hours of voluntary/extracurricular teaching have you attended on research methodology? (none, less than 2 hours, 2-5 hours, 5-10 hours, >10 hours)
19. What form did your research teaching take? (lecture, seminar, tutorial, e-learning, other)

Responses to following questions: (strongly disagree, disagree, neutral, agree, strongly agree)

1. I have had sufficient training in research methodology at medical school
2. I have had sufficient opportunity to participate in research at medical school

## Part Two. Your Perceptions of Research.

### Your Perceptions of Medical Research

Please answer the following questions with a response from 0-10, where 0 = strongly disagree, 5 = neither agree nor disagree and 10 = strongly agree.

1. I enjoy research
2. I am good at research
3. I am confident conducting research
4. I am experienced at research
5. Research is interesting
6. Research is important
7. Research is difficult
8. Research is best left to scientists and/or senior doctors
9. I would consider being involved in research in the future

### Your Perceptions of Systematic Reviews

Please answer the following questions with a response from 0-10, where 0 = strongly disagree, 5 = neither agree nor disagree and 10 = strongly agree.

1. I have experience conducting systematic reviews
2. I am confident with the theory of a systematic review
3. I am confident with the practicalities of conducing a systematic review
4. I am excited about being involved in a systematic review
5. Being involved in a systematic review will improve my understanding and experience of research
6. A collaborative systematic review is a good way to build my skills, experience and confidence before working independently in the future

On a scale of 0-10 with 0 representing the lowest and 10 the highest score please rate your knowledge of each of the following stages of a systematic review

1. Question formulation
2. Development of a search strategy
3. Development of inclusion and exclusion criteria
4. Title and abstract screening
5. Full text screening
6. Risk of bias assessment
7. Development of an extraction template
8. Data extraction
9. Data synthesis
10. Data interpretation
11. Manuscript writing
12. Presentation skills

On a scale of 0-10 with 0 representing the lowest and 10 the highest score please rate your confidence with each of the following stages of a systematic review

1. Question formulation
2. Development of a search strategy
3. Development of inclusion and exclusion criteria
4. Title and abstract screening
5. Full text screening
6. Risk of bias assessment
7. Development of an extraction template
8. Data extraction
9. Data synthesis
10. Data interpretation
11. Manuscript writing
12. Presentation skills

On a scale of 0-10 with 0 representing the lowest and 10 the highest score please rate your experience of working on each of the following stages of a systematic review

1. Question formulation
2. Development of a search strategy
3. Development of inclusion and exclusion criteria
4. Title and abstract screening
5. Full text screening
6. Risk of bias assessment
7. Development of an extraction template
8. Data extraction
9. Data synthesis
10. Data interpretation
11. Manuscript writing
12. Presentation skills

# Survey 2 - Mid-screening questionnaire

## Your Perceptions of Medical Research

Please answer the following questions with a response from 0-10, where 0 = strongly disagree, 5 = neither agree nor disagree and 10 = strongly agree.

1. I enjoy research
2. I am good at research
3. I am confident conducting research
4. I am experienced at research
5. Research is interesting
6. Research is important
7. Research is difficult
8. Research is best left to scientists and/or senior doctors
9. I would consider being involved in research in the future

## Your Perceptions of the Collaborative Systematic Review Project

Please answer the following questions with a response from 0-10, where 0 = strongly disagree, 5 = neither agree nor disagree and 10 = strongly agree.

1. I have experience conducting systematic reviews
2. I am confident with the theory of a systematic review
3. I am confident with the practicalities of conducing a systematic review
4. I am excited about being involved in a systematic review
5. Being involved in a systematic review is improving my understanding and experience of research
6. I am satisfied with the level of guidance I am receiving
7. I am enjoying being involved in the systematic review
8. My research skills are improving as a result of being involved in the review
9. My confidence in my ability to conduct research is improving as a result of being involved in the review
10. I could not gain this understanding of research from passive learning e.g. textbook or lecture
11. I am mainly using the desktop version of Rayyan to screen
12. I am mainly using the mobile version of Rayyan to screen
13. I am using the mobile and desktop versions of Rayyan equally
14. The process is easier than I expected
15. The process is more time consuming than I expected
16. This initiative is a useful experience to build my skills, experience and confidence before conducting a systematic review on my own
17. I would recommend the initiative to other students wishing to be involved in research

On a scale of 0-10 with 0 representing the lowest and 10 the highest score please rate your knowledge of each of the following stages of a systematic review

1. Question formulation
2. Development of a search strategy
3. Development of inclusion and exclusion criteria
4. Title and abstract screening
5. Full text screening
6. Risk of bias assessment
7. Development of an extraction template
8. Data extraction
9. Data synthesis
10. Data interpretation
11. Manuscript writing
12. Presentation skills

On a scale of 0-10 with 0 representing the lowest and 10 the highest score please rate your confidence with each of the following stages of a systematic review

1. Question formulation
2. Development of a search strategy
3. Development of inclusion and exclusion criteria
4. Title and abstract screening
5. Full text screening
6. Risk of bias assessment
7. Development of an extraction template
8. Data extraction
9. Data synthesis
10. Data interpretation
11. Manuscript writing
12. Presentation skills

On a scale of 0-10 with 0 representing the lowest and 10 the highest score please rate your experience of working on each of the following stages of a systematic review

1. Question formulation
2. Development of a search strategy
3. Development of inclusion and exclusion criteria
4. Title and abstract screening
5. Full text screening
6. Risk of bias assessment
7. Development of an extraction template
8. Data extraction
9. Data synthesis
10. Data interpretation
11. Manuscript writing
12. Presentation skills

# Survey 3 - Post-screening questionnaire

## Your Perceptions of Medical Research

Please answer the following questions with a response from 0-10, where 0 = strongly disagree, 5 = neither agree nor disagree and 10 = strongly agree.

1. I enjoy research
2. I am good at research
3. I am confident conducting research
4. I am experienced at research
5. Research is interesting
6. Research is important
7. Research is difficult
8. Research is best left to scientists and/or senior doctors
9. I would consider being involved in research in the future

## Your Perceptions of the Collaborative Systematic Review Project

Please answer the following questions with a response from 0-10, where 0 = strongly disagree, 5 = neither agree nor disagree and 10 = strongly agree.

1. I have experience conducting systematic reviews
2. I am confident with the theory of a systematic review
3. I am confident with the practicalities of conducing a systematic review
4. I am satisfied with the level of guidance I am receiving
5. I am enjoying being involved in the systematic review
6. My research skills are improving as a result of being involved in the review
7. My confidence in my ability to conduct research is improving as a result of being involved in the review
8. I could not gain this understanding of research from passive learning e.g. textbook or lecture
9. The process is easier than I expected
10. The process is more time consuming than I expected
11. This initiative is a useful experience to build my skills, experience and confidence before conducting a systematic review on my own
12. This collaborative systematic review improved my research experience
13. My understanding of research methodology improved as a result of being part of this review
14. Being involved in this research has made me more likely to do research in future
15. Being involved in this research has made me more likely to do myelopathy research in the future

Please answer the following questions with a response of either ‘Yes’ or ‘No’.

1. Would you have preferred to be involved in all stages of the review?
2. Overall, was the experience worthwhile?

On a scale of 0-10 with 0 representing the lowest and 10 the highest score please rate your knowledge of each of the following stages of a systematic review

1. Question formulation
2. Development of a search strategy
3. Development of inclusion and exclusion criteria
4. Title and abstract screening
5. Full text screening
6. Risk of bias assessment
7. Development of an extraction template
8. Data extraction
9. Data synthesis
10. Data interpretation
11. Manuscript writing
12. Presentation skills

On a scale of 0-10 with 0 representing the lowest and 10 the highest score please rate your confidence with each of the following stages of a systematic review

1. Question formulation
2. Development of a search strategy
3. Development of inclusion and exclusion criteria
4. Title and abstract screening
5. Full text screening
6. Risk of bias assessment
7. Development of an extraction template
8. Data extraction
9. Data synthesis
10. Data interpretation
11. Manuscript writing
12. Presentation skills

On a scale of 0-10 with 0 representing the lowest and 10 the highest score please rate your experience of working on each of the following stages of a systematic review

1. Question formulation
2. Development of a search strategy
3. Development of inclusion and exclusion criteria
4. Title and abstract screening
5. Full text screening
6. Risk of bias assessment
7. Development of an extraction template
8. Data extraction
9. Data synthesis
10. Data interpretation
11. Manuscript writing
12. Presentation skills

# Student Demographics

|  | Response | Frequency |
| --- | --- | --- |
|  |  |  |
| **Sex** |  |  |
|  | Male | 8 (57.1 %) |
|  | Female | 6 (42.9%) |
| **Age** |  |  |
|  | 20 | 1 (7.1%) |
|  | 21 | 4 (28.6%) |
|  | 22 | 0 (0%) |
|  | 23 | 3 (21.4%) |
|  | 24 | 1 (7.1%) |
|  | 25 | 0 (0%) |
|  | 26 | 3 (21.4%) |
|  | 27 | 0 (0%) |
|  | 28 | 2 (14.3%) |
| **Year of Study** |  |  |
|  | Year 3 | 2 (14.3%) |
|  | Year 4 | 7 (50%) |
|  | Year 5 | 2 (14.3%) |
|  | Year 6 | 2 (14.3%) |
|  | Foundation Year 1 | 1 (7.1%) |
| **Previous Degrees** |  |  |
|  | BSc or BA | 5 (35.7%) |
|  | MSc | 3 (21.4%) |
| **Specialties of Interest** |  |  |
|  | Neurology or Neurosurgery | 10 (71.4%) |
|  | Paediatrics | 2 (14.3%) |
|  | Undecided | 3 (21.4%) |
| **Is research necessary to secure a training post in your desired specialty?** |  |  |
|  | Yes | 10 (71.4%) |
|  | No | 1 (7.1%) |
|  | Not sure | 3 (21.4%) |
|  |  |  |
| **Previously been an author of a PubMed-indexed systematic review** |  |  |
|  | Yes | 2 (14.3%) |
|  | No | 12 (85.7%) |
| **Previously published a first-author publication in a Pubmed-indexed journal** |  |  |
|  | Yes | 3 (21.4%) |
|  | No | 11 (78.6%) |
| **Previously published a non-first-author publication in a Pubmed-indexed journal** |  |  |
|  | Yes | 3 (21.4%) |
|  | No | 11 (78.6%) |
| **Previously published a publication in non-Pubmed-indexed journals e.g. student journals** |  |  |
|  | Yes | 3 (21.4%) |
|  | No | 11 (78.6%) |
| **Previous laboratory research experience?** |  |  |
|  |  | 11 (78.6%) |
|  |  | 3 (21.4%) |
| **Previous clinical research experience?** |  |  |
|  | Yes | 9 (64.3%) |
|  | No | 5 (35.7%) |
| **Previously presented research at national or international conferences** |  |  |
|  | Yes | 8 (57.1%) |
|  | No | 6 (42.9%) |
| **Previously presented research at regional, local or student conferences** |  |  |
|  | Yes | 9 (64.3%) |
|  | No | 5 (35.7%) |
|  |  |  |
| **Hours of mandatory teaching on research methodology received at University?** |  |  |
|  | None | 0 (0%) |
|  | < 2 hrs | 4 (28.6%) |
|  | 2-5 hrs | 3 (21.4%) |
|  | 5-10 hrs | 3 (21.4%) |
|  | >10 hrs | 4 (28.6%) |
| **Hours of voluntary/extra-curricular teaching on research methodology attended at University?** |  |  |
|  | None | 4 (28.6%) |
|  | < 2 hrs | 3 (21.4%) |
|  | 2-5 hrs | 4 (28.6%) |
|  | 5-10 hrs | 1 (7.1%) |
|  | >10 hrs | 2 (14.3%) |
| **Form of research teaching** |  |  |
|  | Lecture | 6 (42.9%) |
|  | Seminar | 3 (21.4%) |
|  | Tutorial | 2 (14.3%) |
|  | Other | 3 (21.4%) |
| **To what extent do you agree with the following statements: I have had sufficient training in research methodology at medical school** |  |  |
|  | Strongly Agree | 0 (0%) |
|  | Agree | 4 (28.6%) |
|  | Neutral | 5 (35.7%) |
|  | Disagree | 3 (21.4%) |
|  | Strongly Disagree | 2 (14.3%) |
| **To what extent do you agree with the following statements: I have had sufficient opportunity to participate in research at medical school** |  |  |
|  | Strongly Agree | 2 (14.3%) |
|  | Agree | 5 (35.7%) |
|  | Neutral | 3 (21.4%) |
|  | Disagree | 3 (21.4%) |
|  | Strongly Disagree | 1 (7.1%) |
